# Developing Machine Learning Algorithms on Routinely Collected Administrative Health Data - Lessons from Ontario, Canada.

**DOI:** 10.23889/ijpds.v7i3.1851

**Published:** 2022-08-25

**Authors:** Vinyas Harish, Mathieu Ravaut, Seung Eun Yi, Jahir Gutierrez, Hamed Sadeghi, Kin Kwan Leung, Tristan Watson, Kathy Kornas, Tomi Poutanen, Maksims Volkovs, Laura Rosella

**Affiliations:** 1 University of Toronto; 2 Layer 6 AI

There has been considerable growth in the development of machine learning models for clinical applications; however, less attention has been paid to applications at the health systems level. Here, we survey recent models developed using provincial administrative health data holdings in Ontario, Canada to synthesize key learnings across use cases.

We have developed four models in the areas of diabetes incidence and complications, hospitalization due to ambulatory care sensitive conditions, and hospitalization due to SARS-CoV-2 infection. Our team was highly multidisciplinary with expertise across clinical medicine, administrative health data, epidemiology, and computer science. We used a “sliding window” approach to aggregate healthcare events across multiple health administrative data sets chronologically and map them dynamically onto a patient timeline. Tree-based algorithms, specifically gradient boosted decision trees, are well suited for the underlying tabular structure of administrative data and were used for each prediction task.

Our models achieved excellent discrimination, measured by the area under the receiver operating characteristic curve, between 0.77-0.85 at prediction windows between 30 days and 3 years in advance. They were also well-calibrated, both in-the-large and in population subgroups such as older adults, those living in rural areas, and the materially deprived. Measures of feature importance revealed that our models were leveraging predictors across administrative datasets (e.g. demographics, interactions with the healthcare system, medications) in intuitive and defensible ways. Finally, we demonstrated the utility of our models with “recall at top k” metrics - for example, the top 1% of patients predicted at risk of diabetes complications represented a cost of over $400 million to the healthcare system.

We identify three key learnings needed for the successful application of machine learning methods to health administrative data: synergy between nature of training data and intended algorithm use, adherence to methodological best practices for rigour and transparency, and multidisciplinary teams with expertise across data provenance, methodological approach, and impact assessment.

